# Mitochondrial Dysfunction in Circulating Blood Cells and Biological Aging: A Review of Mechanisms and Evidence

**DOI:** 10.3390/biom16070972

**Published:** 2026-07-01

**Authors:** Abdullah M. AlShahrani, S Rehan Ahmad

**Affiliations:** 1 Department of Basic Medical Science, College of Applied Medical Sciences, King Khalid University (KKU), Abha 62561, Saudi Arabia; 2 Hiralal Mazumdar Memorial College for Women, West Bengal State University, Kolkata 700035, West Bengal, India; 3 Department of Biotechnology, UCSI University, Cheras, Kuala Lumpur 56000, Malaysia

**Keywords:** mitochondrial dysfunction, biological aging, mtDNA copy number, mitochondrial membrane potential, cell-free mitochondrial DNA, peripheral blood mononuclear cells, aging biomarkers, oxidative stress, inflammaging, clinical translation

## Abstract

Chronological age tells us how long a person has lived—but not how well. Two individuals of the same age can differ dramatically in their cellular health, disease risk, and functional capacity. This gap between calendar age and biological age has driven growing interest in biomarkers that reflect true cellular aging rather than years lived. Mitochondria sit at the heart of this problem. Far more than cellular power plants, these organelles govern energy production, oxidative stress, immune signaling, and programmed cell death. As the body ages, mitochondria deteriorate in consistent and measurable ways—and crucially, these changes can be detected in circulating blood cells, offering a minimally invasive window into the body’s biological age. This narrative review synthesizes two decades of research (2005–2025) on three blood-based mitochondrial markers: mitochondrial DNA copy number (mtDNA-CN) in peripheral blood mononuclear cells, mitochondrial membrane potential (MMP), and cell-free mitochondrial DNA (cf-mtDNA) in plasma. Across 68 carefully selected studies, we evaluate the strength, consistency, and clinical relevance of each marker, alongside their associations with cardiovascular disease, metabolic dysfunction, cognitive decline, and mortality. The evidence is promising but still maturing. Significant methodological variation across studies limits direct comparisons, and robust prospective outcome data remain limited. We propose a four-phase framework for responsible clinical translation and identify specific research investments needed—from measurement standardization to large cohort studies and intervention trials—before these markers can responsibly inform patient care.

## 1. Introduction

If you were asked to guess how healthy someone is, you might start by asking their age, and for good reason—age is one of the strongest predictors of disease risk and mortality that medicine has ever identified. Yet any experienced clinician knows that two patients sitting in the same waiting room, both aged 65, can be in radically different health states. One may run marathons; the other may struggle to climb stairs. One may have the metabolic profile of a 45-year-old; the other may show signs of organ aging well beyond their years. Chronological age, measured simply as the number of years since birth, is a useful but blunt instrument. It tells us how long a person has been alive, but not how quickly or how well their body’s machinery has aged at the cellular level.

This recognition has fueled an extraordinary surge of scientific interest in what researchers call ‘biological aging’—the actual rate of cellular and molecular deterioration that drives disease, disability, and death, independent of the calendar. Over the past three decades, the search for reliable biological aging biomarkers—measurable signals that accurately reflect the true biological state of an individual’s tissues—has become one of the most active frontiers of biomedical science. Such markers could, in principle, allow clinicians to identify who is aging faster than expected, to guide the timing and intensity of preventive interventions, and to track whether treatments are actually slowing the aging process at its roots.

At the center of this search are mitochondria. These remarkable double-membraned organelles—present in nearly all nucleated cells of the human body—are far more than simple ‘powerhouses.’ They are dynamic, multifunctional organelles that govern not only the production of adenosine triphosphate (ATP), the universal cellular energy currency, but also regulate the generation and detoxification of reactive oxygen species (ROS), coordinate cellular responses to stress and nutrient availability, initiate and control programmed cell death (apoptosis), buffer intracellular calcium, and orchestrate inflammatory signaling pathways. Mitochondria are, in the most literal sense, the metabolic hubs upon which cellular life depends.

With advancing age, mitochondria deteriorate in a characteristic and well-documented pattern. Their ability to produce ATP declines. The rate at which they generate damaging ROS increases relative to their antioxidant defenses. Their quality control system—the process called mitophagy, by which damaged mitochondria are selectively identified and destroyed—becomes less efficient. The mitochondrial genome (mtDNA) accumulates mutations and loses copies. And the inflammatory signals generated by damaged mitochondria begin to chronically activate the immune system in ways that accelerate tissue aging throughout the body. These changes have been documented across virtually every major tissue type, from skeletal muscle and brain to liver and heart.

A key discovery—both scientifically important and practically significant—is that mitochondrial aging is not confined to deep, difficult-to-access tissues. It is also measurable in circulating blood cells. Peripheral blood mononuclear cells (PBMCs)—the lymphocytes and monocytes that make up the white cell fraction of blood—maintain functional, intact mitochondria. Changes in these cells’ mitochondrial health with aging appear to reflect, at least partially, the broader pattern of mitochondrial deterioration occurring throughout the body. Because blood can be sampled easily, repeatedly, and with minimal patient burden, blood-based mitochondrial measurements offer a potentially powerful strategy for tracking biological aging at the population and individual levels.

Three blood-based mitochondrial markers have attracted the most sustained research attention. Mitochondrial DNA copy number (mtDNA-CN) in PBMCs—the number of copies of the mitochondrial genome present per cell—has been measured in multiple large epidemiological cohorts and consistently declines with age, with associations documented for cardiovascular disease, metabolic syndrome, cognitive impairment, and all-cause mortality. Mitochondrial membrane potential (MMP)—the electrochemical gradient across the inner mitochondrial membrane that drives ATP synthesis—can be measured in living blood cells by flow cytometry and shows progressive deterioration from early adulthood into old age. Cell-free mitochondrial DNA (cf-mtDNA) in plasma—fragments of mitochondrial DNA released from damaged or dying cells into the bloodstream—rises with age and has emerged as a potential circulating inflammatory signal capable of activating the innate immune system.

A question worth addressing at the outset is why these three markers form the focus of this review. Mitochondrial biology offers many measurable parameters—including mitophagy flux, reactive oxygen species production, and cardiolipin oxidation—each reflecting a genuine dimension of mitochondrial health. The reason mtDNA-CN, MMP, and cf-mtDNA were prioritized is that all three can be measured in peripheral blood using protocols scalable to clinical and epidemiological settings without tissue biopsies or highly specialized equipment. mtDNA-CN is quantifiable from stored DNA by qPCR; MMP is assessable in living PBMCs by flow cytometry; and cf-mtDNA is detectable in plasma by standard nucleic acid extraction. By contrast, mitophagy flux requires specialized assay systems unsuited to large-scale human studies, and ROS quantification in blood cells suffers from pre-analytical instability that complicates standardization. These three markers thus represent the subset of blood-based mitochondrial measurements with the greatest current translational potential—not the full picture of mitochondrial aging, but the most accessible window into it that the field presently offers.

This review synthesizes two decades of research on these markers, drawing on studies published between 2005 and 2025. It is deliberately structured to do several things simultaneously: explain the underlying biology in accessible terms; evaluate the quality of the clinical evidence honestly and rigorously; compare blood mitochondrial markers with other established aging biomarkers; assess the evidence for interventions that target mitochondrial health; and identify with precision the research investments that would be needed to translate this promising science into clinical medicine. Throughout, the review adopts an explicitly cautious and evidence-grounded tone—because the history of medicine is full of promising biomarkers that never made it to clinical practice, and because patients and clinicians deserve an honest assessment of where the science currently stands.

This is not a systematic review or meta-analysis. It is a narrative synthesis of a rapidly evolving, methodologically heterogeneous literature, with all the strengths and limitations that this design entails. Wherever possible, quantitative effect sizes are provided and evidence quality is explicitly labeled. The goal is not to advocate for blood mitochondrial biomarkers, but to provide the clearest possible picture of what the evidence shows.

## 2. Materials and Methods

Before presenting the evidence, it is worth explaining how this review was constructed and why certain methodological choices were made. The literature on blood mitochondrial biomarkers is technically heterogeneous in ways that have real consequences for how studies should be interpreted—and those consequences shaped every aspect of how we searched, selected, and evaluated the evidence described in the sections that follow.

### 2.1. Study Design and Rationale for Narrative Review

This manuscript is a narrative review, which was chosen deliberately over a systematic review or meta-analysis. The reasons for this choice are methodologically important. The measurement methods for the three principal blood mitochondrial biomarkers—mtDNA copy number, mitochondrial membrane potential, and cell-free mtDNA—are not sufficiently standardized across laboratories to permit meaningful pooling of quantitative data from different research groups. Study populations, cell isolation protocols, choice of reference genes, flow cytometry parameters, and analytical platforms differ substantially between institutions and research teams. These differences mean that even well-conducted studies measuring the same biological phenomenon produce results that cannot be directly compared numerically. Under these conditions, a meta-analysis would generate statistically precise but biologically misleading conclusions.

A narrative review approach allows us to identify patterns across studies, note areas of consistency and inconsistency, integrate mechanistic information with epidemiological data, and make qualitative judgments about evidence quality—while being explicit about the limitations of that approach. Readers should interpret the syntheses presented in this review as thematic overviews and directional assessments rather than exhaustive, statistically combined evidence summaries.

### 2.2. Literature Search Strategy

A comprehensive literature search was conducted across four major scientific databases: PubMed/MEDLINE, ScienceDirect, Web of Science (Core Collection), and Google Scholar. Searches covered publications between January 2005 and December 2025, with selected pre-2005 publications included to provide mechanistic and historical context. The following MeSH term and keyword combinations were used: (‘mitochondrial dysfunction’ OR ‘mitochondrial function’) AND (‘blood cells’ OR ‘peripheral blood’ OR ‘PBMC’ OR ‘lymphocytes’ OR ‘monocytes’ OR ‘platelets’) AND (‘aging’ OR ‘ageing’ OR ‘biological age’ OR ‘biomarkers’). Secondary searches were conducted for specific topics including mtDNA copy number and aging, mitochondrial membrane potential and blood aging, cell-free mitochondrial DNA and plasma aging, mtDNA and cardiovascular disease, mtDNA and cognitive aging, exercise and mitochondria and blood cells, and NAD+ or NMN or NR and mitochondria and aging. Reference lists of identified reviews and high-citation primary studies were manually screened for additional relevant literature.

### 2.3. Study Selection Criteria

Studies were included if they reported original data on at least one mitochondrial marker in human blood cells or plasma, included an age-related analysis (either cross-sectional age comparison or longitudinal follow-up), reported outcomes with sufficient methodological detail to permit quality appraisal, and were published in peer-reviewed journals indexed in PubMed, Scopus, or Web of Science. Studies were excluded if they measured mitochondrial function exclusively in non-blood tissues without parallel blood measurements, if sample sizes were fewer than 15 participants without clear mechanistic justification, if methodological reporting was insufficient, or if they were conference abstracts, editorials, or opinion pieces without original data. A complete list of the 29 full-text articles excluded after final review, along with specific reasons for exclusion, is provided in Supplementary Table S1.

### 2.4. Quality Assessment

While a formal GRADE assessment was not conducted—given the heterogeneous study designs and the absence of a single focused research question amenable to such appraisal—individual studies were informally evaluated using a modified Critical Appraisal Skills Programme (CASP) framework. Studies were assessed on the following dimensions: clarity of the research question, appropriateness of the study design, adequacy of exposure measurement including standardization and blinding, outcome ascertainment quality including prospective versus retrospective design, control of confounding variables, precision of effect estimates with confidence intervals, and generalizability to broader populations. Studies assessed as low quality on three or more dimensions were cited only where higher-quality alternatives were unavailable, with explicit notation of quality concerns.

An estimated 312 records were initially identified across all databases. After removal of duplicates (*n* = 78), screening by title and abstract (*n* = 234 screened), and full-text review (*n* = 97 retrieved), a final corpus of approximately 68 studies formed the basis for the evidence synthesis presented in this review.

### 2.5. Evidence Labeling Framework

Throughout this review, evidence quality is explicitly labeled in tables and text using four categories: High evidence (supported by five or more independent prospective cohorts with *n* > 1000 each and consistent associations with hard clinical outcomes); Moderate evidence (supported by three or more cross-sectional studies with mechanistic support but limited prospective outcome data); Emerging evidence (supported by one or two small studies with limited outcome data); and Low or Insufficient evidence (single study or inconsistent findings across studies). This labeling is intended to help readers distinguish between what is well-established and what remains speculative.

## 3. Mechanistic Foundations of the Three Blood Mitochondrial Markers in Aging

This review is built around three markers: mitochondrial DNA copy number (mtDNA-CN), mitochondrial membrane potential (MMP), and cell-free mitochondrial DNA (cf-mtDNA). All three reflect the same basic process, the gradual decline of mitochondria in circulating blood cells, but each captures a different side of it. Section 3.1 first sets out the biology behind that decline—the failures in energy production, genome maintenance, quality control, and inflammatory signalling that the markers ultimately track—before the three subsections that follow take up each marker on its own: mtDNA-CN (Section 3.2), MMP (Section 3.3), and cf-mtDNA (Section 3.4).

### 3.1. Mitochondrial Dysfunction as a Hallmark of Aging: Shared Mechanistic Context

The idea that mitochondria sit at the center of aging is more than half a century old. It began with Denham Harman’s free radical hypothesis, first proposed in the 1950s and developed through the 1970s, which held that the oxygen chemistry of normal metabolism inevitably produces reactive molecules that damage DNA, proteins, and membranes over a lifetime. Because mitochondria are both the main site of oxygen use and the principal source of these reactive species, they are at once the cell’s engine and a steady source of its wear. The hypothesis gained experimental weight decades later: Trifunovic and colleagues showed in 2004 that mice engineered to accumulate mitochondrial DNA (mtDNA) mutations aged prematurely across multiple organs, and Kujoth and colleagues reported in 2005 that such mutations drive cell death in metabolically active tissues, pointing to a causal rather than merely correlative role. Modern accounts have moved well beyond the original free radical idea. Mitochondria are now understood as active signaling hubs that report the cell’s metabolic and stress state to the nucleus, to neighboring cells, and to the immune system, and their dysfunction is formally recognized as one of the twelve interconnected hallmarks of aging [1,2].

The first of these shared mechanisms is oxidative imbalance. As electrons pass along the electron transport chain, a small fraction escape and react with oxygen to form superoxide, which is then converted to hydrogen peroxide and cleared by antioxidant enzymes such as superoxide dismutase 2, catalase, and glutathione peroxidase. In young cells this defense comfortably keeps pace. With age it does not: antioxidant capacity falls, Complexes I and III become less efficient, and oxidation of cardiolipin destabilizes the chain, so the balance tips toward a chronic excess of reactive species and the cumulative damage that follows. In blood cells the shift is measurable as a clear rise in mitochondrial reactive oxygen species in older adults, tracking with markers of metabolic and inflammatory aging.

A second mechanism is the gradual failure of mitochondrial quality control. Cells normally identify and dispose of damaged mitochondria through mitophagy, a process governed largely by the proteins PINK1 and Parkin: in a healthy organelle PINK1 is continually cleared, whereas in a damaged, depolarized one it builds up and recruits Parkin, which tags the whole mitochondrion for removal [3]. With age this system slows on several fronts at once, as PINK1 and Parkin activity declines and lysosomal and autophagic capacity wanes. Damaged mitochondria therefore linger, continuing to consume resources, generate excess reactive species [4,5], and leak their contents into the cell and beyond—the first step toward the release of mitochondrial material into the circulation that Section 3.4 takes up.

The third mechanism connects the first two to the immune system. Aging is accompanied by a chronic, low-grade, sterile inflammation, termed inflammaging by Franceschi and Campisi, now recognized as a major driver of cardiovascular disease, type 2 diabetes, neurodegeneration, and cancer in later life [6]. Mitochondria are central to it. When damaged organelles spill their contents, those fragments—mtDNA, cardiolipin, and formylated peptides among them—are read by innate immune receptors that evolved to detect microbes and cannot tell self from foreign, producing a persistent, simmering activation of inflammatory pathways that the body never fully switches off. How circulating mitochondrial DNA sustains this signaling is examined in Section 3.4.

**Figure 1:**
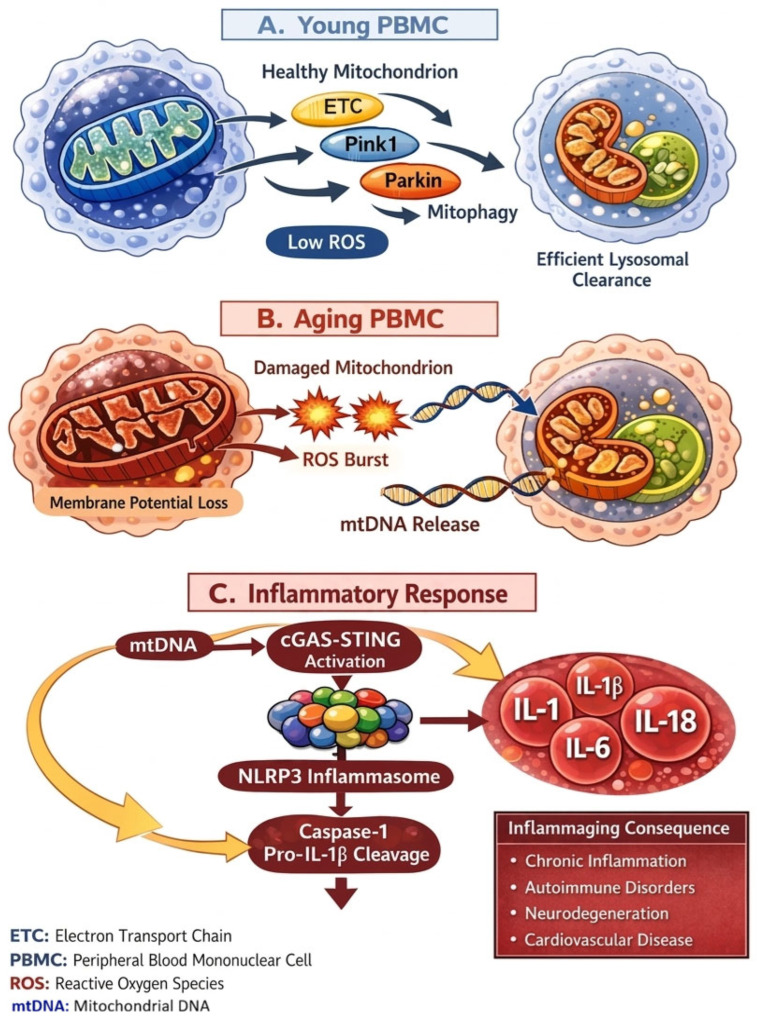
Mitochondrial aging cascade in blood cells.

Taken together, these processes tell a single, coherent story, and it leads directly to the three markers at the heart of this review. The weakening of the electron transport chain and its proton gradient is what mitochondrial membrane potential captures in living cells; the accumulation of mtDNA damage and the failure to hold copy number steady is what mtDNA-CN reflects; and the breakdown of quality control, ending in membrane rupture and the release of mtDNA into the blood, is what cf-mtDNA measures. Figure 1 draws these threads together in a single blood cell, following it from the efficient mitochondria and brisk quality control of youth to an aged state in which oxidative damage, a falling membrane potential, and stalled mitophagy converge on mtDNA release and the inflammatory cascade that fuels inflammaging. The three subsections that follow take each in turn.

### 3.2. Mitochondrial DNA Copy Number (mtDNA-CN): Biological Basis

Of the three markers, mitochondrial DNA copy number asks the simplest question: how many copies of the mitochondrial genome does a cell still hold? Unlike most of the cell, mitochondria carry their own DNA, and they carry it in many copies at once. mtDNA-CN measures that overall abundance. Because abundance reflects how well a cell has built and maintained its mitochondria over a lifetime, it reads as a marker of mitochondrial capacity rather than of any one damaged molecule.

The genome being counted is a circular, double-stranded molecule of approximately 16,569 base pairs that encodes 37 genes: 13 proteins forming essential subunits of the electron transport chain and ATP synthase, 22 transfer RNAs, and 2 ribosomal RNAs needed for mitochondrial protein synthesis. The remaining roughly 1500 mitochondrial proteins are encoded in the nucleus, made in the cytoplasm, and imported. Normal function therefore depends on two separate genomes staying coordinated, which is itself a built-in point of vulnerability.

Compared with nuclear DNA, mitochondrial DNA is more vulnerable to damage for several interconnected reasons. It lacks the protective histone coating that packages nuclear DNA into chromatin, leaving it relatively exposed. It sits in the matrix, close to the inner membrane where ROS are generated, which puts it in a target-rich position for oxidative attack [7,8]. And although it can carry out base excision repair, it lacks the nucleotide excision repair and homologous recombination pathways available to the nucleus, so its toolkit for correcting certain classes of damage is narrower. Each mitochondrion also holds multiple copies of mtDNA, typically 2 to 10, and each cell holds hundreds to thousands of mitochondria, giving hundreds to thousands of copies per cell—a redundancy called polyploidy [9], which lets mutant and normal molecules coexist within one cell as heteroplasmy.

Two distinct things happen to this genome with age, and telling them apart is what makes sense of the marker. The first is a loss of quality. The most studied lesion, 8-hydroxydeoxyguanosine (8-OHdG), forms when guanine is oxidized by ROS, and its level in mtDNA rises sharply with age—by some estimates ten- to twentyfold higher than in nuclear DNA. Point mutations and small deletions also accumulate, particularly in the control region (D-loop) that governs replication and transcription and at hotspot sequences prone to oxidative attack [10]. These changes degrade electron transport chain assembly, impair oxidative phosphorylation, and amplify ROS production in a self-reinforcing cycle [11]. The second change, and the one mtDNA-CN actually captures, is a loss of quantity: the total number of mtDNA copies per cell declines with age [12], independently of how many mutations have accrued.

Why the copies are lost is not fully settled. The likely contributors are reduced activity of PGC-1α (peroxisome proliferator-activated receptor gamma coactivator 1-alpha) and TFAM (mitochondrial transcription factor A), the principal regulators of mitochondrial biogenesis; mitophagy clearing damaged mitochondria faster than new ones are made; and the replicative aging of immune cell populations. The decline is best documented in peripheral blood mononuclear cells, which is what makes it usable as a blood marker—measuring mtDNA-CN amounts to asking how well a person’s cells have held on to their mitochondrial genome across a lifetime of stress. How much it changes across the lifespan is quantified in Section 4.2, how it is measured in Section 5, and what it predicts clinically in Section 6.

### 3.3. Mitochondrial Membrane Potential (MMP): Biological Basis

Where mtDNA-CN counts the genome, mitochondrial membrane potential measures whether the machinery that genome encodes is actually working. MMP is the voltage held across the inner mitochondrial membrane, and it is produced by the very process that defines a healthy mitochondrion: the flow of electrons down the respiratory chain.

In a young, healthy cell, electrons stripped from glucose, fatty acids, and amino acids pass in sequence through the four complexes of the electron transport chain (ETC) embedded in the inner membrane (Complexes I to IV), ending at Complex IV, where they reduce molecular oxygen to water. As the electrons move, the complexes pump protons out of the matrix into the intermembrane space, building an electrochemical gradient across the membrane. That gradient is the membrane potential, and it is what Complex V (ATP synthase) draws on to make ATP. A strong, stable potential is therefore a sign that the respiratory chain is intact and coupled; a collapsing one is a sign that it is not.

With age, the gradient weakens. As the electron transport chain deteriorates, with Complexes I and III losing efficiency and electron flow turning leakier, fewer protons are pumped for each electron that passes, and the membrane potential progressively depolarizes [13]. Because depolarization is also the signal that marks a mitochondrion for clearance by mitophagy [3], a chronically low potential both reflects damage and contributes to the quality-control failure described in Section 3.1.

This is what makes MMP a useful marker rather than an abstract laboratory value: it is a direct, real-time readout of whether the electron transport chain is doing its job. Unlike mtDNA-CN, which reports on accumulated genomic maintenance, MMP captures the current functional state of the organelle, which is why the two markers are complementary rather than redundant. In blood it can be measured in living, intact cells using potential-sensitive fluorescent dyes; how it is quantified across leukocyte populations is detailed in Section 4.4, the assays and their standardization in Section 5, and its disease associations in Section 6.

### 3.4. Cell-Free Mitochondrial DNA (cf-mtDNA): Biological Basis

The third marker is not measured inside cells at all. Cell-free mitochondrial DNA refers to fragments of the mitochondrial genome that have escaped the cell entirely and circulate freely in the blood. Where mtDNA-CN looks at what a cell has kept and MMP at how well it is running, cf-mtDNA reads what it has spilled—and that spillage carries a strong signal about both mitochondrial damage and the inflammation that accompanies aging.

cf-mtDNA originates in the failure of mitochondrial quality control described earlier. When mitophagy can no longer keep pace with damage, dysfunctional mitochondria accumulate and their outer membranes become prone to permeabilization, releasing mtDNA fragments first into the cytoplasm and, through both cell death and active secretion, eventually into the bloodstream [14,15]. Measuring cf-mtDNA in plasma is therefore not detecting a random byproduct; it captures the downstream consequence of a quality-control system overwhelmed by the accumulated damage of aging. Consistent with this, circulating mtDNA rises with age and tracks with the inflammatory state of older adults [16].

**Figure 2:**
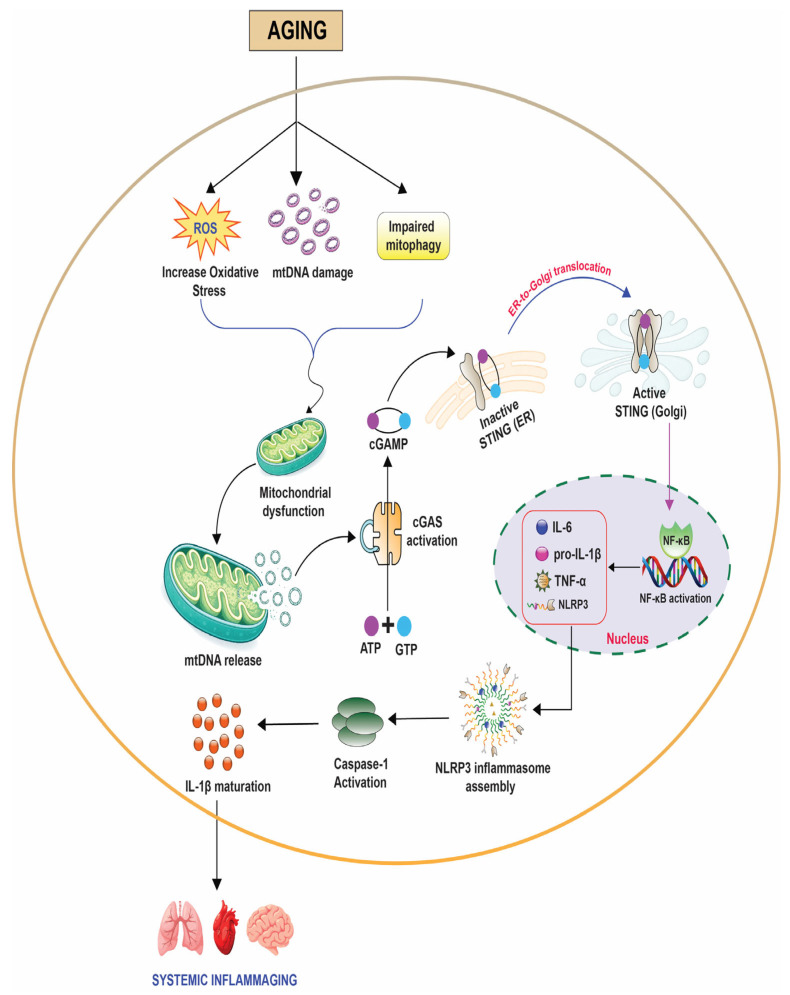
Mitochondrial dysfunction and biological aging pathway in circulating blood cells. Illustrates age-related mitochondrial changes including increased reactive oxygen species (ROS), mtDNA damage and mutation, impaired mitophagy, and reduced ATP production, leading to cellular dysfunction and systemic inflammaging via cGAS-STING and NLRP3 pathways.

What makes cf-mtDNA more than a passive marker of damage is what the immune system does with it. Because mitochondria descend from ancestral bacteria, their DNA still carries bacterial-like features, and the innate immune receptors that evolved to detect microbes cannot tell self-derived mitochondrial DNA from the real thing. Circulating and cytoplasmic mtDNA is therefore read as a danger signal—a damage-associated molecular pattern—through several routes: the cGAS-STING pathway, which senses cytosolic DNA and drives type I interferon and NF-κB-dependent cytokine production [17,18]; the NLRP3 inflammasome, which matures IL-1β and IL-18 [14]; and Toll-like receptor 9 on monocytes and dendritic cells, which amplifies cytokine release [18]. So the very molecule released by failing mitochondria feeds the chronic, low-grade inflammation of aging, making cf-mtDNA at once a marker of damage and a participant in it (Figure 2).

This dual character is what sets cf-mtDNA apart from the other two markers. mtDNA-CN and MMP report on mitochondria still inside cells; cf-mtDNA reports on what has been lost and on the inflammatory consequences of that loss. How its concentration changes across the lifespan is set out in Section 4.3, the assays used to quantify it and their pre-analytical pitfalls in Section 5, and its associations with disease and mortality in Section 6.

## 4. Mitochondrial Changes Across Blood Cell Populations

**Figure 3:**
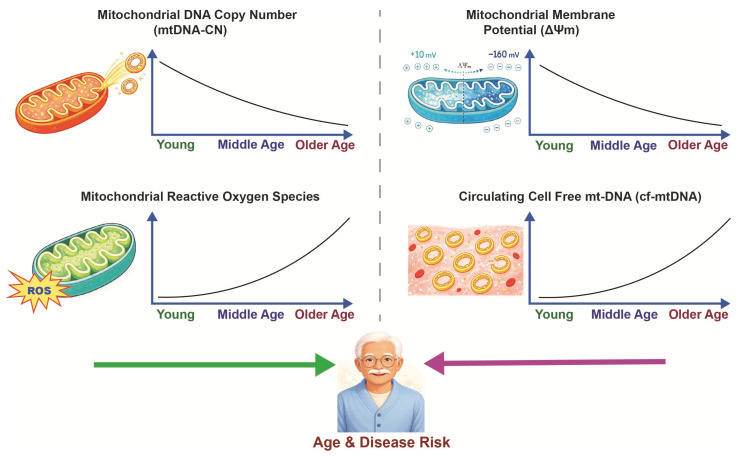
Mitochondrial changes across blood cell populations.

Blood is not a homogeneous tissue. It is a complex mixture of functionally distinct cell populations—red blood cells, platelets, neutrophils, lymphocytes, monocytes, and natural killer cells—each with its own mitochondrial characteristics, metabolic demands, and lifespan. Understanding how aging affects mitochondrial function in each of these populations separately is essential for interpreting blood-based mitochondrial measurements correctly (Figure 3 and Table 1).

### 4.1. Peripheral Blood Mononuclear Cells: The Most Extensively Studied Population

PBMCs—comprising CD3+ T lymphocytes, CD19+ B lymphocytes, CD56+ natural killer (NK) cells, and CD14+ monocytes—represent the most thoroughly characterized blood cell population for mitochondrial aging research. These cells retain functional, intact mitochondria with all four electron transport chain complexes active, making them genuinely useful windows into systemic mitochondrial health. Each PBMC contains between 1 and 10 mitochondria on average, with monocytes harboring higher mitochondrial mass than T cells due to their greater cytoplasmic volume and metabolic activity [19,20].

An important and frequently underappreciated complication is that the cellular composition of PBMCs changes substantially with age—a phenomenon called immunosenescence. Aging is associated with a marked increase in the proportion of terminally differentiated, effector memory T cells (particularly CD8+ TEMRA cells) and a reduction in naive lymphocytes [21]. These terminally differentiated T cells have characteristically lower mitochondrial mass and function compared with naive or central memory T cells. This compositional shift means that some of the apparent decline in aggregate PBMC mitochondrial measurements seen with aging may reflect this cellular reshuffling rather than—or in addition to—genuine deterioration within individual cell types. Most current studies do not correct for this confound, which represents a significant methodological limitation of the existing literature.

### 4.2. Quantitative Data: How Much Does mtDNA Copy Number Actually Change with Age?

The most extensively studied blood mitochondrial marker is mtDNA copy number per PBMC. Understanding the magnitude of this change—and its variability—is essential for evaluating the clinical potential of this marker. The data below represent approximate means from available literature using qPCR-based assays, with the important caveat that values differ substantially by measurement method, reference gene selection, cell isolation protocol, and laboratory (Table 2).

Several important interpretive points emerge from this data. First, the overlap between age groups is enormous. The coefficients of variation of 40 to 60 percent mean that individual-level variation greatly exceeds age-group differences. A single blood mtDNA measurement in an individual cannot reliably indicate ‘biological old age’ relative to their chronological peers. This wide variation has profound implications for clinical use: it means that mtDNA copy number as a standalone test would generate a very high rate of false positives and false negatives if used to classify individuals as biologically aged or not aged.

Second, the annual rate of decline—approximately 2 to 3 percent per year on average—is slow enough that meaningful within-person changes would require longitudinal measurement windows of many years to be reliably detectable above measurement noise. Third, sex differences in mtDNA copy number have been inconsistently reported, with some studies finding higher copy numbers in premenopausal women (possibly reflecting mitochondrial biogenesis effects of estrogen signaling through ERα) while others find no sex effect after covariate adjustment [23]. Fourth, most large datasets were drawn from European-ancestry populations in North America or Northern Europe, leaving substantial uncertainty about whether the normative ranges presented above apply to populations of Asian, African, or South Asian ancestry.

### 4.3. Platelets and Cell-Free mtDNA: Emerging but Understudied Compartments

Platelets—the small, anucleate cell fragments derived from megakaryocytes—retain functional mitochondria, unlike erythrocytes (red blood cells), which shed their mitochondria during final maturation. Platelet mitochondrial function is measurable using the Seahorse XF analyzer (Agilent Technologies, Santa Clara, CA, USA), which quantifies oxygen consumption rate as a proxy for oxidative phosphorylation capacity [22]. Studies comparing platelets from elderly versus young individuals have documented reduced basal oxygen consumption rate, lower maximal respiratory capacity (the maximum rate of oxygen consumption achievable under uncoupled conditions), and reduced spare respiratory capacity (the reserve capacity available to meet sudden increases in ATP demand). These changes have been proposed as potential contributors to age-related platelet hyperactivation and increased thrombotic risk, since mitochondrial dysfunction alters thromboxane synthesis pathways and ADP-mediated platelet aggregation. However, platelet mitochondrial studies remain small (typically fewer than 50 participants), lack standardization, and have not yet been linked to hard cardiovascular endpoints in prospective analyses.

Cell-free mitochondrial DNA in plasma occupies a conceptually important but empirically undercharacterized position in this field. As detailed in the mechanistic section above, cf-mtDNA functions as a circulating DAMP capable of activating cGAS-STING, TLR9, and NLRP3 inflammasome pathways on immune cells, thereby generating and perpetuating systemic inflammation [16]. Circulating cf-mtDNA levels rise with advancing age, with frailty, with acute illness including sepsis and myocardial infarction, and with major surgical procedures. A study of ICU patients demonstrated that high plasma cf-mtDNA on admission was significantly associated with 28-day mortality, suggesting potential utility as an acute prognostic marker in critical illness. However, in the aging context, cf-mtDNA research remains largely descriptive, with no established normal reference ranges for age and sex, no longitudinal outcome studies, and no consensus on whether plasma or serum, or specific subfractions (vesicular versus free-floating), should be preferentially measured.

### 4.4. Mitochondrial Membrane Potential in PBMCs: What It Measures and Why It Matters

Of the three biomarkers reviewed here, mitochondrial membrane potential is perhaps the most directly functional. While mtDNA-CN tells us something about the genomic integrity of mitochondria and cf-mtDNA tells us about what has already been lost or released, MMP tells us whether the mitochondria that are present are actually working—right now, in living cells. The electrochemical gradient across the inner mitochondrial membrane, generated by proton pumping through the electron transport chain, is the immediate driving force for ATP synthesis. When this gradient weakens, energy production falters, and the cell moves closer to apoptotic activation.

In circulating PBMCs, MMP is measured by flow cytometry using fluorescent dyes that accumulate in mitochondria in proportion to the strength of the electrochemical gradient. The most widely used are JC-1, valued for its ratiometric readout that partially corrects for variation in mitochondrial mass between cells, and TMRE, which offers greater sensitivity but requires more careful optimization. Across studies using these approaches, MMP in PBMCs declines by approximately 20 to 35 percent between early adulthood and old age—a magnitude of change that is biologically meaningful and broadly comparable to the mtDNA-CN decline documented in Section 4.2.

One practical constraint that distinguishes MMP from mtDNA-CN measurement deserves emphasis. Because MMP is a property of living, energized mitochondria, it can only be assessed in fresh cells. Blood must be processed within four to six hours of collection, and any delays or temperature fluctuations introduce artefactual depolarization that cannot be corrected afterwards. This pre-analytical vulnerability is one of the main reasons MMP has been harder to standardize across laboratories than mtDNA-CN, which can be reliably measured from frozen DNA long after collection.

The consequences of MMP decline in aging immune cells go beyond simply reduced ATP availability. A depolarized inner membrane lowers the threshold for mitochondrial permeability transition, priming cells toward apoptosis. It impairs calcium buffering, disrupting T cell activation. And it increases the likelihood of outer membrane permeabilization—contributing directly to the release of cf-mtDNA into the circulation, as described in Section 4.3. MMP decline is therefore not an isolated finding but a convergence point connecting reduced energy capacity, apoptotic vulnerability, and inflammatory signaling in a single measurable parameter.

## 5. Measurement Methods, Costs, and Standardization Status

**Figure 4:**
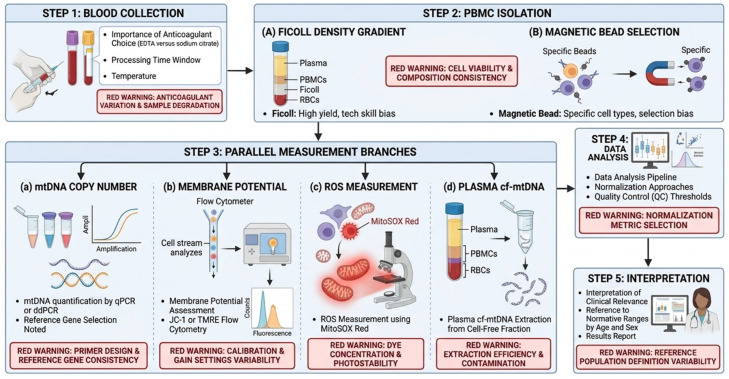
Measurement methods, costs, and standardization status.

One of the most important—and most underappreciated—aspects of blood mitochondrial biomarker research is the substantial technical heterogeneity in how these markers are measured across different laboratories. This is not merely a technical inconvenience; it is the single most important barrier to clinical translation and the principal reason why multi-center studies have been difficult to interpret and why results from different research groups often cannot be meaningfully compared [24] (Table 3 and Figure 4).

### The Standardization Crisis: The Fundamental Barrier to Clinical Translation

The absence of internationally harmonized measurement protocols for blood mitochondrial biomarkers is not a minor technical detail but a fundamental structural barrier to clinical implementation [25,26].

When different laboratories analyze identically prepared PBMC samples and report results that vary by two- to threefold, the data from different studies cannot be pooled, compared, or integrated—precisely the kind of large, multi-center analyses that would be needed to generate the prospective evidence required for clinical validation.

To understand the magnitude of this problem, consider just one source of variability: the choice of nuclear DNA reference gene for normalizing mtDNA copy number measurements by qPCR. Different laboratories use different reference genes—GAPDH, beta-actin (ACTB), RNaseP, TERT, [27] or others—each of which has different copy numbers per cell, different expression patterns across cell types, different susceptibility to pseudogene interference, and different stability under various storage conditions. The choice of reference gene alone can shift the reported mtDNA copy number value by 20 to 50 percent in the same biological sample. Multiply this by the additional variability introduced by different PBMC isolation protocols (Ficoll density gradient versus magnetic bead selection versus whole blood lysis), different fluorescent dyes for membrane potential measurement, different processing times between blood draw and analysis, and different flow cytometry gating strategies, and the result is a literature in which cross-study comparisons are deeply unreliable.

A useful parallel is the early history of telomere length measurement as a biological aging biomarker. In the 1990s and early 2000s, telomere research faced essentially the same problem: different laboratories measured telomere length using different methods (Southern blotting, qPCR, FISH), producing results that could not be compared across studies. The telomere research community eventually invested approximately a decade in systematic standardization work—establishing reference DNA materials, calculating inter-laboratory coefficients of variation, publishing harmonized protocols, and creating the Telomere Standardization Consortium (STNC) approach—before the field reached a level of methodological consistency that enabled reliable multi-center and meta-analytic work. The blood mitochondrial biomarker field has not yet seriously begun this process. Until it does, the accumulation of additional studies will not resolve the fundamental problem of cross-study incomparability.

## 6. Association with Age-Related Disease and Mortality: Evidence from Prospective Studies

**Figure 5:**
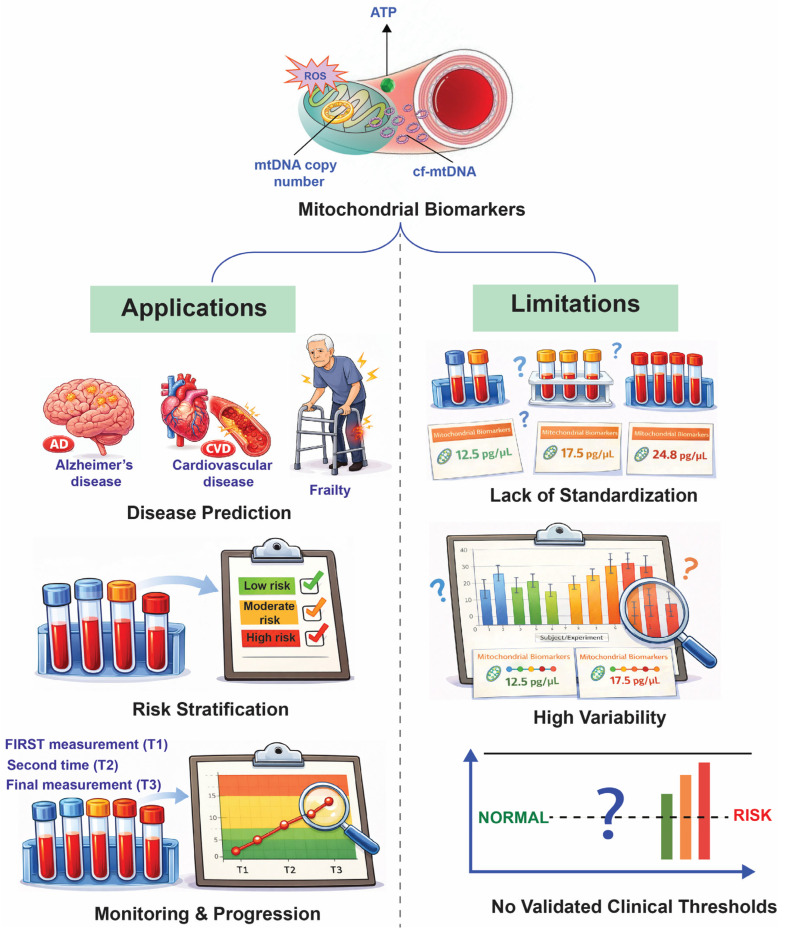
Clinical potential and limitations of blood mitochondrial biomarkers in aging. This shows the application of mitochondrial biomarkers in disease prediction, risk stratification, and monitoring, alongside key limitations such as lack of standardization, high variability, and absence of validated clinical thresholds.

The central question for clinical medicine is not whether mitochondrial function changes with age—it does—but whether those changes predict clinically meaningful outcomes: heart attacks, strokes, dementia, diabetes, disability, and death. The following sections summarize what the prospective epidemiological evidence shows, with explicit attention to effect sizes, confidence intervals, and the limitations of each study design (Table 4 & Figure 5).

### 6.1. Cardiovascular Disease: The Strongest and Most Replicated Evidence Base

Of the three biomarkers reviewed in this paper, the cardiovascular disease evidence base rests most heavily on mtDNA-CN, which has been measured in several large prospective cohorts with hard clinical endpoints. cf-mtDNA has also been examined in the acute coronary syndrome setting, though the data here are far more preliminary. MMP, by contrast, has not yet been studied in relation to cardiovascular outcomes in prospective designs, and its role in this context remains largely mechanistic and inferential.

The association between blood mitochondrial markers—particularly mtDNA copy number—and cardiovascular disease risk represents the strongest and most consistently replicated finding in this field. Multiple large prospective cohort studies conducted in independent populations have reported statistically significant associations with cardiovascular endpoints, providing a degree of evidence for this specific association that is substantially stronger than for any other outcome examined in this literature.

In a sub-study of the Women’s Health Initiative (WHI)—one of the largest and most rigorous women’s health research programs ever conducted—lower tertile mtDNA copy number at baseline was associated with significantly increased cardiovascular mortality over six years of follow-up (adjusted hazard ratio 1.82, 95% confidence interval 1.21–2.74; *p* = 0.004) [28], after statistical adjustment for age, smoking status, body mass index, and prevalent cardiovascular disease. This effect size is clinically meaningful: an 82 percent increase in relative risk of cardiovascular death is comparable to the incremental risk conferred by mildly elevated LDL cholesterol or a moderate increase in systolic blood pressure.

In the Multi-Ethnic Study of Atherosclerosis (MESA)—a carefully designed prospective cohort that deliberately recruited participants across four ethnic groups—each one-standard-deviation lower mtDNA copy number was associated with a 23 percent higher risk of incident coronary artery disease (adjusted HR 1.23, 95% CI 1.05–1.44), with broadly consistent effects across ethnic subgroups [29]. A European biobank consortium study found that low mtDNA copy number predicted incident heart failure over a median follow-up of 8.5 years (adjusted HR 1.54, 95% CI 1.18–2.01) [30]. Effect sizes vary across studies—ranging from HR 1.2 to 2.5—reflecting genuine heterogeneity attributable to differences in study populations, cardiovascular endpoint definitions, covariate adjustment strategies, and measurement protocols.

A general but important conclusion from this literature is that low blood mtDNA copy number confers approximately 40 to 80 percent increased relative risk of major cardiovascular events. However, several critical caveats must accompany this conclusion. First, these are relative risk estimates; the absolute risk contribution for any individual is substantially smaller and depends on baseline cardiovascular risk. Second, the degree to which mtDNA copy number provides predictive information independent of established cardiovascular risk factors—blood pressure, cholesterol, smoking, diabetes, and family history—has been inconsistently assessed and may be modest [14,30,32].

Third, no randomized controlled trial has tested whether improving mtDNA copy number reduces cardiovascular events, which means the association, however consistent, does not prove that mtDNA copy number is a modifiable risk factor.

Elevated plasma cf-mtDNA has been documented in acute myocardial infarction, correlating positively with peak troponin levels and infarct size estimated by cardiac MRI. However, these data come from small case-control studies with 20 to 80 participants per group, and it remains unclear whether cf-mtDNA provides prognostic information beyond established cardiac biomarkers [31] such as high-sensitivity troponin, BNP, or CRP in the acute coronary syndrome setting 6.1; Table 4.

### 6.2. Cognitive Aging and Neurodegeneration: Promising but Largely Cross-Sectional

The cognitive aging literature has focused primarily on mtDNA-CN, which has been measured in PBMC populations across several cross-sectional studies of Alzheimer’s disease and general cognitive performance. cf-mtDNA has attracted growing interest as a potential driver of neuroinflammation through cGAS-STING activation, though longitudinal outcome data remain limited. MMP has not been systematically studied in relation to cognitive aging in blood-based designs, representing a notable gap in the existing literature.

The human brain accounts for approximately 20 percent of total body energy expenditure despite constituting only about 2 percent of body mass—a reflection of the extraordinary energy demands of neural computation, synaptic transmission, and membrane potential maintenance. Neurons are critically dependent on continuous, efficient mitochondrial function, and their long axons impose particular demands on mitochondrial transport and local energy supply. The hypothesis that systemic mitochondrial dysfunction, as reflected in blood cells, might predict or contribute to cognitive aging has therefore attracted substantial scientific interest.

Cross-sectional studies consistently find lower PBMC mtDNA copy number in patients with Alzheimer’s disease compared with age-matched cognitively normal controls, with typical differences in the range of 15 to 30 percent [33]. Lower mtDNA copy number has also been associated with worse performance on cognitive tests measuring memory, executive function, and processing speed in population-based surveys of older adults [35]. These associations are biologically plausible and statistically robust in cross-sectional designs.

However, causal interpretation of these cross-sectional findings is severely constrained. Patients with established Alzheimer’s disease may show lower blood mtDNA copy number as a consequence [18] of the systemic metabolic and inflammatory changes that accompany neurodegeneration—including reduced physical activity, medication effects, and altered immune function—rather than as a preceding causal factor [18,34]. The few longitudinal studies that have measured baseline blood mitochondrial markers and followed participants to ascertain subsequent cognitive outcomes have yielded inconsistent results: some find a predictive association, others do not, and most suffer from short follow-up periods and small sample sizes [35]. The field currently lacks the prospective data to determine whether blood mitochondrial measurements could serve as early cognitive aging biomarkers—a question of enormous practical importance given the absence of disease-modifying treatments for Alzheimer’s disease 6.2; Table 4.

### 6.3. Metabolic Syndrome and Type 2 Diabetes

Metabolic syndrome and type 2 diabetes are the disease contexts in which all three biomarkers have received at least some attention. mtDNA-CN is consistently lower in individuals with diabetes and metabolic syndrome. MMP impairment has been documented in PBMCs from diabetic patients, reflecting the bidirectional relationship between mitochondrial dysfunction and insulin resistance. And elevated mitochondrial ROS—closely linked to MMP deterioration—correlates with HbA1c levels across cross-sectional studies.

Individuals with type 2 diabetes mellitus and metabolic syndrome consistently show evidence of accelerated mitochondrial aging in blood cells: lower mtDNA copy number, elevated mitochondrial ROS (correlating with HbA1c levels, r ≈ 0.45–0.60 in cross-sectional studies), and impaired mitochondrial membrane potential. The mechanistic relationship is genuinely bidirectional and involves multiple reinforcing pathways. Mitochondrial dysfunction impairs insulin signaling downstream [36] of the insulin receptor through ROS-mediated serine phosphorylation of insulin receptor substrate proteins, reducing glucose uptake in muscle and adipose tissue. Conversely, hyperglycemia itself drives glycation-mediated mitochondrial damage and advanced glycation end-product (AGE) formation, which impairs ETC complex function and increases mitochondrial oxidative stress [33].

One aspect of mitochondrial dysfunction in the metabolic disease context that deserves specific mention is the mitochondrial permeability transition pore—a channel in the inner mitochondrial membrane whose sustained opening leads to membrane potential collapse and cell death. Under normal conditions the mPTP opens transiently and closes. But under the chronic hyperglycemic and hyperlipidemic conditions that define metabolic syndrome and type 2 diabetes, the threshold for sustained opening is dramatically lowered—elevated mitochondrial calcium, excess ROS, and oxidative modification of pore-forming components all conspire to keep it open. Recent evidence from diabetic mouse myocardium and vascular endothelial cells exposed to hyperglycemic stress has confirmed that spontaneous mPTP opening is an active driver of mitochondrial dysfunction in cardiometabolic tissues, not merely a downstream consequence [41,42]. Critically for the biomarker framework of this review, sustained mPTP opening directly causes MMP collapse and accelerates mtDNA release into the circulation—meaning that elevated cf-mtDNA and impaired MMP in metabolic disease patients may partly reflect mPTP-driven damage superimposed on the background trajectory of biological aging.

Whether blood mitochondrial measurements add clinically useful predictive information beyond established metabolic markers—HbA1c, fasting glucose, HOMA-IR, waist circumference, and lipid panels—remains undemonstrated. The correlations documented in cross-sectional studies, while biologically informative, do not in themselves establish that adding mtDNA copy number to existing metabolic risk scores would meaningfully improve risk classification or guide clinical decisions 6.3; Table 4.

### 6.4. Frailty and All-Cause Mortality: Consistent Associations, Uncertain Mechanisms

In the frailty and mortality literature, mtDNA-CN and cf-mtDNA have both been studied with meaningful results, while MMP data in this context remain sparse. mtDNA-CN has prospective mortality data from three independent cohorts, and cf-mtDNA shows strong cross-sectional associations with frailty status. The absence of MMP data in longitudinal frailty studies is a gap worth noting, given the strong biological rationale for its involvement in the sarcopenia and immune senescence that underlie the frailty syndrome.

The association of lower PBMC mtDNA copy number with all-cause mortality has been reported in three independent prospective cohort studies with follow-up periods of 5 to 12 years, with adjusted hazard ratios ranging from 1.35 to 2.05 across studies. Frailty—a clinical syndrome characterized by muscle weakness, weight loss, exhaustion, slow walking speed, and low physical activity, operationalized either by Fried’s phenotypic criteria or the Frailty Index—shows strong cross-sectional associations with both low mtDNA copy number and elevated plasma cf-mtDNA levels [27,37,38].

The biological plausibility of these associations is high. Frailty is characterized by sarcopenia, immune dysregulation, chronic low-grade inflammation, and impaired metabolic efficiency—all of which have established mitochondrial components. The mechanistic model would suggest that accelerated mitochondrial deterioration contributes to sarcopenia through impaired mitochondrial energy supply in skeletal muscle and increased apoptotic signaling, contributes to immune senescence through altered lymphocyte and monocyte mitochondrial function, and drives the chronic inflammation of frailty through DAMP-mediated innate immune activation. Whether blood mitochondrial measurements in clinically robust middle-aged individuals predict frailty onset many years later remains untested, and this is one of the most important questions for future prospective studies 6.4; Table 4.

## 7. Interventions Targeting Mitochondrial Function: A Rigorous Evidence Assessment

The clinical value of any biomarker ultimately depends on whether it can be moved—whether interventions that improve the marker also improve health. This section evaluates the evidence for interventions that have been shown to affect the three blood mitochondrial markers at the center of this review: mtDNA-CN, MMP, and cf-mtDNA, paying close attention to study quality and the critical distinction between biomarker effects and clinical outcome effects (Table 5). For each intervention we ask the same questions: which of the three markers does it affect, by how much, and—critically—does improving those markers actually translate into better outcomes for patients? As the evidence below shows, the answers to the first two questions are often encouraging, while the answer to the third remains, for most interventions, frustratingly incomplete.

### 7.1. Exercise: Established Health Benefits, Unproven Mitochondrial Mediation

Aerobic exercise is the single best-supported intervention for improving blood mitochondrial markers. Multiple well-designed randomized controlled trials have demonstrated that structured aerobic exercise—particularly moderate-intensity continuous training at the equivalent of 150 min per week and high-intensity interval training (HIIT) protocols—produces consistent and meaningful improvements in blood mitochondrial measurements across diverse populations. Exercise activates PGC-1α through AMPK and SIRT1 signaling pathways, stimulating mitochondrial biogenesis. It upregulates mitochondrial antioxidant enzymes including SOD2 and catalase. It enhances mitophagy flux through LC3-II-dependent mechanisms. And it increases mtDNA copy number in PBMCs by 15 to 30 percent over 12 to 24 weeks of regular training [43,44].

Exercise also improves mitochondrial membrane potential in PBMCs, reflecting genuine restoration of ETC function rather than simply an increase in mitochondrial number—though its effects on circulating cf-mtDNA levels have not been consistently measured across intervention studies, representing a straightforward gap that future exercise trials should address.

However, a critical and frequently overlooked distinction must be maintained. The cardiovascular, metabolic, cognitive, and all-cause mortality benefits of regular aerobic exercise are among the most robustly established facts in all of clinical medicine—demonstrated by decades of randomized trial and epidemiological evidence that predates the mitochondrial biomarker literature entirely. The question of whether these health benefits are specifically mediated through exercise’s effects on blood mitochondrial markers—or whether those markers are simply epiphenomenal correlates of general physiological improvement—has never been tested. No randomized trial has been designed with the explicit goal of testing whether exercise’s benefits are mediated by its mitochondrial effects, and no mediation analysis in existing exercise trial data has demonstrated that mitochondrial biomarker improvements explain a significant fraction of the clinical benefit [44].

Additionally, exercise intervention studies in the mitochondrial context have been conducted predominantly in self-selected, motivated, non-smoking, relatively healthy volunteers—not in the sedentary, multi-morbid, medication-taking populations who bear the greatest burden of age-related disease and who would be the targets of clinical intervention. Real-world exercise adherence rates at recommended doses are low—typically 15 to 30 percent in general population surveys [45]—meaning that the theoretical benefit observed in trial participants cannot be straightforwardly extrapolated to the broader population.

### 7.2. NAD+ Precursors: Mechanistically Compelling, Clinically Unproven

The scientific rationale for using NAD+ precursors—primarily nicotinamide mononucleotide (NMN) and nicotinamide riboside (NR)—to support mitochondrial health in aging is one of the most intellectually compelling in contemporary aging pharmacology. NAD+ is an essential cofactor for three key classes of enzymes: sirtuins (particularly SIRT1 and SIRT3), which deacetylate and thereby activate numerous mitochondrial and metabolic proteins; PARP-1, which competes with sirtuins for NAD+ in response to DNA damage; and CD38, which consumes NAD+ in immune signaling contexts. Because intracellular NAD+ levels decline substantially with age in multiple tissues—including blood cells—the rationale for supplementing NAD+ precursors to restore declining levels is biologically sensible [46].

In terms of the three biomarkers specifically, NAD+ precursors have shown the most consistent effects on MMP and mitochondrial biogenesis markers, modest and inconsistent effects on mtDNA-CN, and virtually no systematic data on circulating cf-mtDNA—a gap that is surprising given the strong theoretical link between NAD+ depletion, mitophagy impairment, and mtDNA release.

Phase I and early Phase II clinical trials in older adults, typically enrolling [47,48] 20 to 70 participants for 8 to 12 weeks, have reported acceptable safety profiles and improvements in selected mitochondrial biomarkers including NAD+ levels, mitochondrial biogenesis gene expression, and in some studies, mitochondrial membrane potential and oxygen consumption rate. In one carefully conducted Phase II trial, Elhassan and colleagues found that NR supplementation raised the skeletal muscle NAD+ metabolome in older men, yet it produced no measurable improvement in mitochondrial function, and blood mitochondrial markers were not examined [47].

However, the field has not converged on the optimal compound (NMN versus NR versus other precursors), optimal dose (a range from 100 to 2000 mg per day has been tested with inconsistent results), optimal duration, the most responsive target population, or whether any biomarker improvements persist after supplementation is discontinued [48]. Critically, no Phase III randomized controlled trial with pre-specified hard clinical endpoints—cardiovascular events, incident disability, cognitive decline, or mortality—has been completed for NMN or NR at any dose or duration. Until this evidentiary gap is filled, NAD+ precursors cannot responsibly be recommended for clinical use based on mitochondrial biomarker considerations.

### 7.3. Urolithin A: The Most Promising Single Pharmacological Agent

Urolithin A is a polyphenol-derived metabolite produced by intestinal microbiota through the metabolism of dietary ellagic acid and ellagitannins found in pomegranates, walnuts, and berries [49]. Its mitochondrial relevance lies in its demonstrated ability to activate mitophagy through PINK1/Parkin-independent mechanisms—enhancing selective removal of damaged mitochondria and improving net mitochondrial quality at the cellular level. By enhancing mitochondrial quality control, urolithin A would theoretically reduce the pool of damaged mitochondria most likely to release mtDNA into the circulation—making cf-mtDNA a particularly logical biomarker endpoint for future urolithin A trials, alongside MMP, neither of which was a primary outcome in the published Phase I/IIa studies.

This mechanistic action directly addresses one of the key age-related defects in mitochondrial quality control described earlier in this review.

A pivotal Phase I trial by Andreux and colleagues, published in Nature Metabolism in 2019 [50], enrolled 66 sedentary older adults and demonstrated dose-dependent improvements in skeletal muscle mitochondrial function markers—including acylcarnitine profiles and cardiolipin species consistent with improved mitochondrial health—and improvements in oxygen consumption rate in muscle biopsies, with an excellent tolerability profile at all doses tested. A subsequent Phase IIa trial reported similar findings with emphasis on mitophagy biomarkers. These are genuinely encouraging early-phase results.

However, important limitations apply. A single Phase I/IIa trial in sedentary older adults, with no clinical outcome follow-up and no long-term safety data, does not constitute evidence of efficacy in the clinical sense [51,52]. The substantial individual variability in intestinal microbiome capacity to produce urolithin A from dietary precursors—estimated to affect 30 to 40 percent of individuals who have low or absent urolithin A-producing capacity—means that standardized supplemental urolithin A would be required to reliably achieve therapeutic levels across a diverse patient population. Long-term safety data beyond four months are absent, and the optimal dose for older, medically complex individuals—as opposed to healthy, sedentary research volunteers—is unknown.

## 8. Discussion

### 8.1. Synthesis: What the Evidence Actually Shows

The past two decades of research on blood mitochondrial biomarkers of biological aging have produced a body of evidence that is scientifically rich but clinically incomplete. The overarching picture is one of mechanistic clarity combined with substantial translational uncertainty. On the mechanistic side, there is essentially no doubt that mitochondrial dysfunction is a genuine, well-characterized, and biologically important component of the aging process—one of twelve recognized hallmarks of aging in the most authoritative current framework. The downstream consequences of mitochondrial deterioration—reduced ATP production, increased oxidative stress, impaired quality control, and inflammatory DAMP signaling—are real, measurable, and relevant to multiple age-related diseases.

On the translational side, however, the picture is considerably more complex. Among the three primary blood mitochondrial markers, mtDNA copy number in PBMCs has the strongest evidence base. Its associations with cardiovascular disease, all-cause mortality, and frailty have been replicated across multiple large prospective cohorts with effect sizes comparable to established clinical risk factors. Yet even this best-supported marker falls short of what would be required for clinical implementation: measurement protocols are not standardized, reference ranges across ethnic groups and sexes are inadequately characterized, the magnitude of association is insufficient to meaningfully reclassify cardiovascular risk for individual patients without combination with other markers, and no intervention trial has demonstrated that improving mtDNA copy number reduces clinical events.

Mitochondrial membrane potential data are moderate in quality [53]. The biological rationale is strong—a depolarized inner mitochondrial membrane directly reflects reduced capacity for ATP synthesis and predicts apoptotic vulnerability—but the evidence base consists predominantly of cross-sectional studies without prospective outcome validation. Cell-free mtDNA remains the most preliminary of the three markers, theoretically compelling as both a disease biomarker and an inflammatory mediator but empirically undercharacterized, with no validated clinical reference ranges and no prospective outcome data in aging populations [54].

### 8.2. AI-Assisted Drafting: A Transparency Note

In the interest of full scholarly transparency, the authors note that sections of this manuscript were drafted with the assistance of an AI language model and subsequently reviewed, verified, and revised by human authors for factual accuracy, clinical interpretation, and scientific judgment. All quantitative data, citations, and clinical conclusions were verified against primary published sources by the authoring team. AI assistance was used for structural drafting and language editing only; all scientific interpretations and evidence quality assessments reflect the independent judgment of the human authors. This disclosure is made consistent with emerging standards for AI-assisted scientific authorship, which require explicit disclosure of the nature and extent of AI involvement in manuscript preparation.

### 8.3. Comparison with Other Biological Aging Biomarkers

Blood mitochondrial markers must be understood within the context of a rapidly growing ecosystem of biological aging biomarkers, each measuring different aspects of the multidimensional aging process. The most clinically advanced competing class of biomarkers—epigenetic clocks—provides a useful comparison point.

Epigenetic clocks, particularly GrimAge (developed by Lu, Levine, Horvath and colleagues) and PhenoAge, are algorithms trained on DNA methylation patterns at hundreds to thousands of CpG sites in blood-derived DNA. GrimAge has demonstrated robust, independent associations with mortality, incident disease burden, and healthspan across multiple large longitudinal cohorts [55], and its predictive accuracy substantially exceeds that of individual mitochondrial markers reported in the same populations. Epigenetic clocks have achieved a degree of technical standardization—with commercially available assays producing comparable results across laboratories [55]—that blood mitochondrial markers have not. However, epigenetic clocks do not directly measure mitochondrial function, energy metabolism, or the inflammatory consequences of mitochondrial damage; they capture an integrative methylation signature that reflects aging across many biological systems simultaneously. Whether blood mitochondrial markers and epigenetic clocks provide independent predictive information—or whether one subsumes the other—has not been rigorously tested in head-to-head comparative studies in the same populations.

Leukocyte telomere length—the oldest and most extensively studied biological aging biomarker—is associated with cardiovascular disease risk, cancer incidence, and mortality [56], and has achieved a degree of measurement standardization through the STNC approach. However, leukocyte telomere length primarily reflects the replicative history of lymphocyte populations and is confounded by immune cell composition changes with aging; it does not directly measure mitochondrial function or energetic capacity. Circulating inflammatory markers—particularly CRP, IL-6, and TNF-α—are cheap, widely available, and predict cardiovascular disease and mortality [57], but are non-specific indicators of systemic inflammation without mechanistic specificity to mitochondrial deterioration.

A synthetic assessment: blood mitochondrial markers offer mechanistic specificity to energy metabolism and mitochondrial quality that more distal markers lack, and they are demonstrably responsive to interventions like exercise. Epigenetic clocks currently offer superior predictive accuracy and better standardization [55]. Telomere length provides complementary information about replicative aging [56]. Inflammatory markers offer broader systemic information at lower cost [57]. The optimal strategy for aging biomarker implementation is likely a multi-marker approach that integrates mitochondrial, epigenetic, inflammatory, and functional measures into composite risk scores—but this approach requires the same prospective validation and methodological harmonization work that individual markers currently lack [40].

### 8.4. Critical Limitations: A Subsectioned Analysis

#### 8.4.1. The Absence of Standardized Measurement Protocols

The most fundamental and most immediately actionable barrier to the clinical implementation of blood mitochondrial biomarkers is the complete absence of internationally harmonized measurement protocols. This is the critical difference between where this field stands today and where the telomere and epigenetic clock fields stand after decades of sustained methodological investment. Without standardized protocols, studies conducted in different laboratories using different measurement approaches cannot be combined, and the large prospective cohort evidence that clinical validation requires cannot be generated. This problem is remediable—standardization is a technical and organizational challenge, not a fundamental biological limitation—but it requires deliberate, coordinated, and funded effort by the scientific community.

#### 8.4.2. The Dominance of Cross-Sectional Study Designs

The overwhelming majority of studies documenting age-related changes in blood mitochondrial markers are cross-sectional: they compare older individuals with younger individuals at a single point in time. While this design efficiently demonstrates that mitochondrial markers differ between age groups, it cannot establish whether mitochondrial decline precedes disease onset, the rate of individual decline over time, whether rapid decliners have worse outcomes than slow decliners, or whether interventions that slow the rate of decline improve clinical outcomes. The irreducible requirement for clinical validation is longitudinal studies following individuals for at least ten years with serial mitochondrial measurements and ascertainment of hard outcomes. Such studies are logistically complex and expensive, but they are the only design that can answer the clinical questions that matter.

#### 8.4.3. The Causation Versus Correlation Problem

Perhaps the most intellectually fundamental unresolved question in this field is whether declining mitochondrial function in blood cells drives aging and disease, or whether it is a consequence—a bystander effect—of disease processes or physiological changes that are themselves driven by other primary aging mechanisms. This question of causal directionality has profound implications for clinical use: a biomarker that merely reflects existing disease process would have limited value for early detection or risk stratification, while a biomarker that causally contributes to disease progression would be a genuine therapeutic target. Mendelian randomization studies, which use genetic instruments to test causal relationships, have produced inconsistent results for mitochondrial markers, and the genetic instruments available are currently of limited strength and specificity.

#### 8.4.4. Blood Cell Composition Confounding

As discussed in Section 4, the cellular composition of blood changes substantially with age through immunosenescence. The shift toward terminally differentiated, mitochondria-poor T-cell subsets in aging means that aggregate PBMC mitochondrial measurements in older individuals may reflect compositional shifts rather than—or in addition to—genuine mitochondrial dysfunction within individual cell types. This confound is not typically addressed in existing studies and represents a systematic bias that may inflate the apparent magnitude of age-related mitochondrial decline when measured in unsorted PBMC fractions. Cell-type correction algorithms, analogous to those developed for epigenetic clock analyses to account for blood cell type composition, represent a promising methodological advance that has not yet been developed or validated for mitochondrial measurements.

#### 8.4.5. Limited Population Diversity

The overwhelming majority of blood mitochondrial biomarker studies—including the major prospective cohorts that provide the strongest association evidence—were conducted in populations of predominantly European ancestry residing in high-income countries. Whether the observed associations generalize to Asian, African, Latin American, Middle Eastern, or South Asian populations is unknown. Baseline mtDNA copy number may vary by genetic ancestry; the gut microbiome composition that determines urolithin A production from dietary precursors varies enormously by geography and diet; and the distribution of mitochondrial haplogroups—which affect baseline mitochondrial efficiency—differs substantially between world populations. Any claim to clinical generalizability of these biomarkers requires deliberate replication in diverse populations.

#### 8.4.6. Intervention Evidence Is Preliminary

As detailed in Section 7, no intervention shown to improve blood mitochondrial markers has been tested in a large, long-duration randomized controlled trial with hard pre-specified clinical endpoints. The exercise literature demonstrates mitochondrial biomarker improvements [43] but cannot attribute health outcomes specifically to the mitochondrial effects. Pharmacological approaches—NAD+ precursors, urolithin A, MitoQ, and coenzyme Q10—have promising early-phase biomarker and safety data but are years away from the Phase III evidence that would be required for clinical recommendation. This gap between biomarker modifiability and proven clinical benefit is, ultimately, the most important limitation of the entire field.

## 9. Future Research Directions

The path from the current state of the evidence to clinically validated, actionable blood mitochondrial biomarkers is long but not impossible to map. The following priority areas represent the specific investments that the scientific and funding community would need to make, roughly in the order in which they should be addressed to build a coherent and cumulative evidence base [24].

### 9.1. International Standardization Initiative (Highest Priority)

The establishment of an international working group—comprising mitochondrial biologists, clinical laboratory specialists, epidemiologists, bioinformaticians, and biostatisticians—to develop, validate, and disseminate harmonized protocols for blood mitochondrial biomarker measurement is the single highest priority action in this field. This working group should be modeled explicitly on the precedents set by the Telomere Standardization Consortium and the DNA methylation clock community. Specific deliverables should include: certified reference DNA materials with assigned mtDNA copy number values; harmonized PBMC isolation and storage protocols specifying anticoagulant, processing time windows, and cryopreservation conditions; consensus reference gene selection for qPCR normalization with documented inter-laboratory CV data; standard operating procedures for flow cytometric membrane [56] potential measurement specifying dye, concentration, incubation temperature, instrument settings, and gating strategy; and an international proficiency testing program with published CV targets (goal: inter-laboratory CV below 15 percent on reference samples). Estimated cost: $5 to 10 million over three years. Potential funders: NIH National Institute on Aging, Wellcome Trust, European Research Council, and industry partners with interests in aging medicine.

### 9.2. Large Prospective Cohort Studies

Large prospective cohort studies—each enrolling more than 5000 participants with baseline and serial blood mitochondrial measurements and at least ten years of follow-up with pre-specified hard outcome ascertainment—represent the irreducible requirement for clinical validation. These studies should incorporate biobank infrastructure enabling later sub-studies and nested case-control analyses. They should measure multiple aging biomarkers simultaneously (epigenetic clocks, telomere length, inflammatory markers, proteomics) to permit comparative analyses of relative predictive value [29]. They should be conducted across diverse geographic and ethnic populations. And they should be initiated only after international measurement [58,59] standardization is achieved—otherwise, multi-center data will not be poolable across cohorts. As of 2025, no such studies are funded or underway for blood mitochondrial biomarkers. Estimated cost: $20 to 50 million per cohort over a 15-year lifecycle.

### 9.3. Single-Cell Mitochondrial Phenotyping Methods

The cell composition confounds described in Section 8.4.4 cannot be fully addressed by statistical correction alone; it requires the ability to measure mitochondrial function within specific, immunophenotyped blood cell subsets. Emerging technologies—including mass cytometry (CyTOF), imaging flow cytometry, and [60] single-cell sequencing approaches combined with metabolic measurement—offer the capability to simultaneously measure immunophenotype and mitochondrial parameters in hundreds of individual cells per sample. Development and validation of these cell-type-specific mitochondrial measurement approaches, and their integration with standardized protocols, should be a methodological priority. These technical developments should ideally be completed before large cohort studies are initiated, to enable prospective incorporation of cell-type-corrected measurements from the start.

### 9.4. Intervention Trials with Hard Clinical Endpoints

The field ultimately requires at least one well-powered randomized controlled trial testing a mitochondrial-targeted intervention against a primary endpoint of hard clinical outcomes—cardiovascular events, incident disability, cognitive decline operationalized by validated neuropsychological criteria, or all-cause mortality. The most appropriate intervention for such a trial, based on current evidence, is structured exercise—aerobic plus resistance training—versus enhanced usual care, with embedded mitochondrial biomarker sub-studies designed to test whether biomarker improvement mediates clinical benefit. This would allow simultaneous advancement of intervention evidence and mechanistic understanding [31]. Pharmacological trials of NAD+ precursors or urolithin A should first complete adequately powered Phase II dose-finding and biomarker efficacy studies before proceeding to Phase III clinical event trials. The estimated cost per adequately powered trial of this kind is $15 to 50 million over 5 to 8 years from initiation to primary endpoint.

### 9.5. Population Diversity and Global Generalizability

Deliberate replication of core epidemiological findings—particularly the associations between mtDNA copy number and cardiovascular disease, mortality, and frailty—in racially, geographically, and socioeconomically diverse populations should be an explicit funding priority. Existing global aging cohorts in Asia (including the Chinese Longitudinal Healthy Longevity Survey and the Korean Longitudinal Study of Ageing), sub-Saharan Africa, and Latin America provide established participant networks and outcome infrastructure that could be leveraged for mitochondrial biomarker add-on studies at relatively modest incremental cost. These studies would require adaptation of protocols to local laboratory capabilities—an additional argument for international standardization providing a clear methodological template that can be implemented in resource-variable settings.

### 9.6. Mechanistic Studies: From Association to Causation

Progress on the causation-versus-correlation question will require mechanistic studies that go beyond epidemiological observation. Mendelian randomization analyses using genome-wide association study (GWAS) data for genetic determinants of blood mtDNA copy number represent one approach, though the current lack of strong and specific genetic instruments limits the power of this method. Targeted interventional studies in animal models—using mitochondria-specific CRISPR tools [61], conditional knockout models, and longitudinal biomarker-outcome designs—can provide experimental evidence for causal directionality that cannot be obtained from human observational data. Human tissue studies linking blood mitochondrial measurements to simultaneous assessments in paired muscle or adipose tissue biopsies can clarify whether blood is a reliable surrogate for systemic mitochondrial health [62]. And deep molecular phenotyping using single-cell multi-omics approaches can map the downstream consequences of blood cell mitochondrial dysfunction in ways that may clarify causal pathways [63,64,65].

## 10. Conclusions

Blood mitochondrial biomarkers sit at the intersection of some of the most fundamental questions in aging biology—why do we age, how does cellular energy metabolism deteriorate, and can we measure biological age accurately?—and some of the most important practical challenges in contemporary preventive medicine—how do we identify who is at greatest risk of age-related disease, how do we track biological aging in large populations, and how do we know if anti-aging interventions are working?

The scientific case for pursuing these markers is strong. Mitochondrial dysfunction is a well-established hallmark of aging with deep mechanistic connections to the major chronic diseases of later life—cardiovascular disease, type 2 diabetes, neurodegeneration, and frailty—through pathways that are now well enough understood at the molecular level to be both biologically credible and potentially therapeutically actionable. Blood-based measurement is feasible, minimally invasive, and scalable. The association between low mtDNA copy number and elevated cardiovascular risk in large prospective cohorts is among the most consistent findings in aging biomarker epidemiology. The demonstration that exercise—and potentially emerging pharmacological agents like urolithin A—improves these markers in intervention studies, providing at least circumstantial evidence of biological responsiveness.

But the clinical case for using these markers in current medical practice is weak. The measurement methods are not standardized. The prospective longitudinal evidence, while promising for mtDNA copy number and cardiovascular outcomes, does not yet meet the bar required for clinical biomarker validation. The causation-versus-correlation question is unresolved. The evidence base is heavily skewed toward European-ancestry populations. No intervention has been proven to improve clinical outcomes specifically through its mitochondrial effects. And the regulatory pathway for implementing these markers in clinical workflows is undefined.

The appropriate response to this situation is not pessimism—the scientific foundations are genuinely strong—but structured, funded, and coordinated investment in the specific research programs that would close these gaps. International standardization, large prospective cohorts, mechanistic intervention trials, and deliberate attention to population diversity represent the minimum portfolio of research investments required. With those investments, there is a realistic, if not certain, path to clinical implementation that could meaningfully advance the precision of aging medicine and the targeting of preventive interventions. Without them, blood mitochondrial markers will remain perpetually in the research phase—interesting to scientists but invisible to the patients who might ultimately benefit.

The bottom line is straightforward: these biomarkers are worth investing in, but they are not ready to use. The field’s task for the coming decade is to do the hard, unsexy, but essential work of standardization, validation, and rigorous intervention testing—so that the promise of blood mitochondrial biomarkers can eventually be delivered to patients as responsibly evidence-based clinical medicine.

## Figures and Tables

**Table 1:** Mitochondrial dysfunction across blood cell populations [13].

Blood Cell Type	Key Marker(s)	Change with Age	Clinical Relevance	Evidence Quality
PBMCs (lymphocytes & monocytes)	mtDNA copy number	↓ 30–50% in individuals ≥70 yrs vs. young adults (mean 150–250 vs. 300–400 copies/cell)	CVD, all-cause mortality; adjusted HRs 1.4–2.1 in prospective cohorts	High
PBMCs	Mitochondrial membrane potential (JC-1/TMRE)	Progressive depolarization; ΔΨm declines ~20–35% from age 30–80	Immune exhaustion, apoptotic priming, inflammatory activation	Moderate
PBMCs, Neutrophils	ROS production (MitoSOX)	2–5-fold increase in older individuals; correlates with HbA1c and IL-6	Oxidative disease risk; endothelial dysfunction	Moderate
Plasma (all cells)	Cell-free mtDNA (cf-mtDNA)	Up to 10-fold higher in frail elderly and sepsis; wide inter-individual variation	Frailty, sepsis severity, MI extent; immune activation via cGAS-STING/TLR9	Emerging
Platelets	Oxygen consumption rate (OCR, Seahorse)	Reduced maximal OCR and spare respiratory capacity in elderly	Thrombotic risk, coagulation dysfunction	Low
Red Blood Cells (RBCs)	Not applicable (anucleate, no mitochondria)	N/A	N/A	N/A

**Table 2:** Quantitative summary of mtDNA copy number in PBMCs across the human lifespan [22].

Age Group	Mean mtDNA CN (Copies/Cell)	SD/Range	Approx. Annual Decline Rate	Important Caveats
Young adults (20–30 yrs)	300–400	±50–70	Reference (baseline)	Most data from European-ancestry populations; limited ethnic diversity in existing datasets
Middle age (40–55 yrs)	250–350	±60–80	~2–3% per year	High inter-individual variation (CV 40–60%); sex differences inconsistently reported across studies
Older adults (60–70 yrs)	200–280	±55–90	~2–3% per year	Confounded by medication use, comorbidities; smoking associated with ~10–15% additional decline
Elderly (≥70 yrs)	150–250	±60–100	~2–3% per year (may accelerate in frailty)	Wide variation; frail elderly may show values below 100 copies/cell; extreme outliers documented

Note: CN = copy number; CV = coefficient of variation. Values are approximate means from available literature. Substantial between-laboratory variation exists.

**Table 3:** Blood-based mitochondrial biomarkers: measurement methods, costs, and standardization status.

Biomarker	Measurement Platform	Approx. Cost (USD)	Standardization Status	Key Technical Issues
mtDNA copy number (qPCR)	Quantitative PCR (nuclear DNA:mtDNA ratio)	$50–150	POOR—no international reference material or consensus protocol	Reference gene selection (GAPDH, ACTB, RNaseP) critically affects results; processing temperature sensitivity; nuclear mtDNA pseudogene interference
mtDNA copy number (ddPCR)	Droplet digital PCR	$200–400	POOR—more precise than qPCR but not standardized	Higher precision and absolute quantitation; expensive; limited availability outside research settings; no certified reference materials
Membrane potential (ΔΨm)	Flow cytometry with JC-1 or TMRE fluorescent dyes	$200–500	POOR—dye choice, gating strategy, and viability thresholds vary widely	Results vary 2–3-fold between labs using different dyes; temperature critically affects values; requires fresh cells; must be processed within 4–6 h of blood draw
ROS production (mtROS)	Flow cytometry with MitoSOX Red	$100–300	VERY POOR—no consensus protocol; high background variability	Highly sensitive to cell isolation method, processing time, stimulation conditions; signal quenching if dye concentration too high; non-mitochondrial fluorescence contamination
Cell-free mtDNA (plasma)	ddPCR or qPCR on plasma DNA	$200–400	VERY POOR—no reference ranges; anticoagulant type affects results	Plasma vs. serum gives different values; freeze-thaw cycles degrade signal; vesicular vs. naked (protein-bound) mtDNA fraction not consistently distinguished
Platelet OCR (Seahorse)	Seahorse XF analyzer	$500–2000	VERY POOR—confined to specialized labs	Requires specialized equipment; prohibitive cost for routine use; requires fresh platelets processed within 2 h of blood draw; not scalable

**Table 4:** Association of blood mitochondrial markers with age-related disease and mortality: Disease/clinical problem and key references.

Disease/Clinical Problem	Key Finding/Evidence Summary	References
**6.1 Cardiovascular Disease—Strongest & Most Replicated Evidence Base**		
Cardiovascular mortality	Women’s Health Initiative (WHI) sub-study: lower tertile mtDNA-CN associated with increased CVD mortality over 6 years (adjusted HR 1.82, 95% CI 1.21–2.74; *p* = 0.004)	[28] Ashar et al., J Gerontol A, 2015
Incident coronary artery disease	Multi-Ethnic Study of Atherosclerosis (MESA): each 1-SD lower mtDNA-CN associated with 23% higher risk of incident CAD (adjusted HR 1.23, 95% CI 1.05–1.44); consistent across ethnic subgroups	[29] Ashar et al., JAMA Cardiology, 2017
Incident heart failure	European biobank consortium: low mtDNA-CN predicted incident heart failure over median 8.5-year follow-up (adjusted HR 1.54, 95% CI 1.18–2.01)	[30] Fu et al., QJM, 2025 (meta-analysis)
Acute myocardial infarction—cf-mtDNA	Elevated plasma cf-mtDNA documented in AMI; correlates positively with peak troponin and infarct size on cardiac MRI. Evidence from small case-control studies (n = 20–80 per group)	[31] Bhatt et al., Eur Heart J, 2020 [16] Pinti et al., Eur J Immunol, 2014
Overall CVD risk meta-analysis	Systematic review and meta-analysis of observational studies confirms low mtDNA-CN confers ~40–80% increased relative risk of major cardiovascular events across independent cohorts	[30] Fu et al., QJM, 2025 [32] Yang, Int J Mol Sci, 2025
**6.2 Cognitive Aging & Neurodegeneration—Promising but Largely Cross-Sectional**		
Alzheimer’s disease—mtDNA-CN	Cross-sectional studies consistently show 15–30% lower PBMC mtDNA-CN in AD patients vs. age-matched cognitively normal controls	[33] Picca et al., Antioxidants, 2020 [34] Li et al., J Neuroinflammation, 2025
Cognitive test performance	Lower mtDNA-CN associated with worse performance on memory, executive function, and processing speed tests in population-based surveys of older adults	[35] Nidadavolu et al., Immun Ageing, 2023
cf-mtDNA & neuroinflammation	Circulating cf-mtDNA activates cGAS-STING pathway driving neuroinflammation—mechanistic link to accelerated cognitive aging; longitudinal outcome data remain limited	[34] Li et al., J Neuroinflammation, 2025 [17] Oduro et al., Acta Pharm Sin B, 2022
Longitudinal evidence	Few longitudinal studies available; results inconsistent—some find predictive association, others do not; most limited by short follow-up and small sample sizes	[35] Nidadavolu et al., Immun Ageing, 2023 [18] West et al., Nat Rev Immunol, 2011
**6.3 Metabolic Syndrome & Type 2 Diabetes**		
mtDNA-CN & insulin resistance	Lower mtDNA-CN consistently documented in T2DM and metabolic syndrome; mitochondrial dysfunction impairs insulin signalling via ROS-mediated serine phosphorylation of IRS proteins	[36] Picard & McEwen, Biol Psychiatry, 2018 [12] Bratic & Larsson, J Clin Invest, 2013
Mitochondrial ROS & HbA1c	Elevated mtROS (MitoSOX) correlates with HbA1c levels (r ≈ 0.45–0.60 in cross-sectional studies); hyperglycaemia drives glycation-mediated mitochondrial damage via AGE formation	[33] Picca et al., Antioxidants, 2020 [13] Sun et al., Mol Cell, 2016
Mitochondrial membrane potential	Impaired MMP (ΔΨm) documented in PBMC from T2DM patients; bidirectional relationship between metabolic dysfunction and mitochondrial deterioration well established mechanistically	[24] Brand & Nicholls, Biochem J, 2011 [22] Filograna et al., Nat Rev MCB, 2021
**6.4 Frailty & All-Cause Mortality—Consistent Associations, Uncertain Mechanisms**		
All-cause mortality—prospective cohorts	Association of lower PBMC mtDNA-CN with all-cause mortality reported in three independent prospective cohort studies (follow-up 5–12 years); adjusted HRs ranging from 1.35 to 2.05	[37] Marzetti et al., Curr Opin Clin Nutr, 2026 [38] Mengel-From et al., Mech Ageing Dev, 2014 [27] Mengel-From et al., Hum Genet, 2014
Frailty—mtDNA-CN & cf-mtDNA	Strong cross-sectional associations of frailty (Fried criteria/Frailty Index) with both low PBMC mtDNA-CN and elevated plasma cf-mtDNA levels	[37] Marzetti et al., Curr Opin Clin Nutr, 2026 [39] Picca et al., Rejuvenation Res, 2018
cf-mtDNA—ICU mortality	High plasma cf-mtDNA on ICU admission significantly associated with 28-day mortality (prospective study); cf-mtDNA acts as inflammatory DAMP activating cGAS-STING and TLR9	[40] Nakahira et al., AJRCCM, 2013 [16] Pinti et al., Eur J Immunol, 2014
Sarcopenia & immune senescence	Mechanistic model: mitochondrial deterioration drives sarcopenia (impaired ATP supply, increased apoptotic signalling) and immune senescence (altered lymphocyte/monocyte function) underlying frailty	[37] Marzetti et al., Curr Opin Clin Nutr, 2026 [13] Sun et al., Mol Cell, 2016 [3] Palikaras et al., Nat Cell Biol, 2018

Abbreviations: mtDNA-CN, mitochondrial DNA copy number; PBMC, peripheral blood mononuclear cell; cf-mtDNA, cell-free mitochondrial DNA; CVD, cardiovascular disease; HR, hazard ratio; CI, confidence interval; T2DM, type 2 diabetes mellitus; ROS, reactive oxygen species; MMP/ΔΨm, mitochondrial membrane potential; AGE, advanced glycation end-product; DAMP, damage-associated molecular pattern; AMI, acute myocardial infarction; ICU, intensive care unit; CAD, coronary artery disease; AD, Alzheimer’s disease.

**Table 5:** Interventions targeting blood mitochondrial markers: Differentiated evidence assessment.

Intervention	Evidence Category	Effect on Mitochondrial Markers	Largest RCT (*n*)	Hard Health Outcomes Demonstrated?	Overall Assessment
Aerobic exercise (150 min/week × 12–24 weeks)	ESTABLISHED	↑ mtDNA CN, ↓ ROS, ↑ ΔΨm, ↑ PGC-1α expression	~200 per arm (exercise RCTs); meta-analyses include thousands	Yes—CVD, mortality benefits established independently of mitochondrial markers	Exercise effects on health are robustly established; mitochondrial mediation of health benefits is unproven
NAD+ precursors (NMN, NR; 250–1000 mg/day)	EXPERIMENTAL	↑ NAD+ levels, ↑ biogenesis markers, ↓ ROS in some studies	70 (Phase II)	No—no efficacy data on clinical outcomes in any completed large RCT	Biomarker effects only; optimal dose, duration, target population unknown; Phase III trials absent
MitoQ (mitochondria-targeted antioxidant)	EXPERIMENTAL	↓ mitochondrial ROS, ↓ cf-mtDNA in pilot studies	<50 (pilot RCTs)	No—no evidence from adequately powered trials	Very preliminary; no Phase III evidence; safety data beyond 3 months extremely limited
Urolithin A (500–1000 mg/day)	EXPERIMENTAL	↑ mitophagy, ↑ OCR, improved mitochondrial membrane integrity	~66 (Phase I/IIa)	No—no clinical outcome follow-up in any completed trial	Most promising single pharmacological agent; good tolerability profile; single early-phase trial; no long-term data
Caloric restriction/Mediterranean-type diet	INVESTIGATIONAL	Modest ↑ mtDNA CN; variable effects on ROS and membrane potential	Variable; primarily observational data	Partial—mortality and CVD benefits documented observationally but mitochondrial-mediated benefit unproven	Strong mechanistic rationale; clinical evidence for specifically mitochondrial-mediated benefit absent
Coenzyme Q10 (100–600 mg/day)	EXPERIMENTAL	Modest ↓ oxidative stress markers; variable effects on ETC function	<100 (meta-analyses of small trials)	No—inconsistent effects on hard outcomes in small trials	Mechanistically plausible; evidence weak and inconsistent; no large definitive RCT

## Data Availability

The datasets generated and/or analysed during the current study are available from the corresponding author, S Rehan Ahmad (professor.rehaan@gmail.com), on reasonable request.

## Supplementary Materials

The following supporting information can be downloaded at: https://www.mdpi.com/article/10.3390/biom16070972/s1, Table S1: Full-text articles reviewed but excluded from the final synthesis (n = 29), with reasons for exclusion. Studies were assessed against inclusion/exclusion criteria detailed in Section 2.3 of the main manuscript [66,67,68,69,70,71,72,73,74,75,76,77,78,79,80,81,82,83,84,85,86,87,88,89,90,91,92,93,94].
