# Supplementary material for: Mitochondrial Dysfunction in Circulating Blood Cells and Biological Aging: A Review of Mechanisms and Evidence
Source: Biomolecules. 2026 Jul 1;16(7):972. doi: 10.3390/biom16070972 (PMC13406649; doi:10.3390/biom16070972)
Supplement: Supplementary file 1 [file biomolecules-16-00972-s001.zip › biomolecules-4310337-supplementary.pdf]

Supplementary Table S1 is available online.

Supplementary Table S1

Full-text articles reviewed but excluded from the final synthesis (n = 29), with reasons for exclusion. Studies were assessed against inclusion/exclusion criteria detailed in Section 2.3 of the main manuscript.

No.	Author(s)	Year	Journal	Title	Reason for Exclusion
**1**	Mittelbrunn et al.	2021	Nature Immunology	Hallmarks of T cell aging	Mitochondrial measurements in non-blood tissue only (T cell signaling, no blood-based assay reported)
**2**	Frye et al.	2024	Neurobiology of Disease	Biomarkers of mitochondrial dysfunction in autism spectrum disorder: A systematic review and meta-analysis	Pediatric/neurodevelopmental disease focus — not an aging population study
**3**	Hubens et al.	2022	Mitochondrion	Blood biomarkers for assessment of mitochondrial dysfunction: An expert review	No age-related analysis reported; review of diagnostic methods without aging outcome data
**4**	Huang et al.	2024	Journal of Advanced Research	Lipidomic analysis identifies long-chain acylcarnitine as a target for	No age-related analysis; focused on ischemic disease mechanism without aging biomarker data

No.	Author(s)	Year	Journal	Title	Reason for Exclusion
				ischemic heart disease	
**5**	Hope et al.	2025	Nature Cancer	Age-associated NAD+ decline drives CAR-T cell failure	Mitochondrial measurements in non-blood tissue (tumor microenvironment); no blood aging biomarker reported
**6**	Aili et al.	2024	European Respiratory Journal	LPG 18:0 is a general biomarker of asthma and inhibits differentiation of inflammatory monocytes	No age-related analysis; focused on asthma pathophysiology without aging outcome
**7**	Lu et al.	2024	BMC Medicine	Integrative bioinformatics analysis for identifying mitochondrial-related biomarkers	No age-related analysis reported; bioinformatics study without human blood aging data
**8**	Huang et al.	2026	Journal of Advanced Research	Fibrinogen exacerbates α-synuclein aggregation and mitochondrial dysfunction	No age-related analysis; focused on Parkinson's disease mechanism

No.	Author(s)	Year	Journal	Title	Reason for Exclusion
**9**	Kurien et al.	2025	RMD Open	Mitochondrial dysfunction and fatigue in Sjögren's disease	No age-related analysis; disease-specific study (Sjögren's) without aging biomarker data
**10**	Escrig-Larena et al.	2023	Seminars in Immunology	Mitochondria during T cell aging	Mitochondrial measurements in non-blood tissue; mechanistic review without original blood-based aging data
**11**	Zeng et al.	2025	Science Translational Medicine	The m6A demethylase FTO links TLR7 to mitochondrial oxidation driving lupus pathogenesis	Mitochondrial measurements in non-blood tissue; focused on lupus mechanism without aging analysis
**12**	Pan et al.	2023	Translational Psychiatry	Metabolic features of treatment-refractory major depressive disorder with mitochondrial dysfunction	No age-related analysis; psychiatric disease focus without aging biomarker outcome
**13**	Henske et al.	2023	Cancer Cell	Chromophobe renal cell carcinoma	Mitochondrial measurements in non-blood tissue (renal cancer cells); no blood

No.	Author(s)	Year	Journal	Title	Reason for Exclusion
					aging biomarker reported
**14**	Chen et al.	2024	Clinical Immunology	Progranulin promotes regulatory T cell plasticity by mitochondrial metabolic reprogramming	Mitochondrial measurements in isolated T cells without aging context; no age-related analysis
**15**	Muñoz-Muela et al.	2024	EBioMedicine	HIV-1-DNA/RNA and immunometabolism in monocytes: contribution to cardiovascular comorbidities	No age-related analysis; HIV-specific immunometabolism study
**16**	Nga et al.	2024	Diabetes & Metabolism Journal	T-Cell Senescence in Human Metabolic Diseases	Mitochondrial measurements in non-blood tissue; no independent blood-based aging analysis
**17**	Braganza et al.	2020	Molecular Aspects of Medicine	Blood-based bioenergetics: An emerging translational and clinical tool	No age-related analysis; methodological review of bioenergetics platforms without aging outcome data

No.	Author(s)	Year	Journal	Title	Reason for Exclusion
**18**	Headley et al.	2024	Advanced Science	Mitochondrial Transplantation Promotes Protective Effector and Memory CD8+ T Cell Responses	Mitochondrial measurements in non-blood tissue; mechanistic study without aging biomarker analysis
**19**	Leuner et al.	2012	Molecular Neurobiology	Peripheral mitochondrial dysfunction in Alzheimer's disease: focus on lymphocytes	No age-related analysis; Alzheimer's disease focus without aging population comparison or biomarker outcome
**20**	Abbasifard et al.	2024	Cell Biochemistry and Function	Peripheral blood mononuclear cells show markers of mitochondrial dysfunction in rheumatoid arthritis	No age-related analysis; rheumatoid arthritis-specific study without aging biomarker data
**21**	Singal et al.	2018	Gene Expression	Cellular Abnormalities and Emerging Biomarkers in Alcohol-Associated Liver Disease	No age-related analysis; alcohol-specific liver disease focus

No.	Author(s)	Year	Journal	Title	Reason for Exclusion
**22**	Plata-Gómez et al.	2025	Journal of Experimental Medicine	Age- and diet-instructed metabolic rewiring of the tumor-immune microenvironment	Mitochondrial measurements in tumor microenvironment tissue; no blood-based aging biomarker reported
**23**	Mihaylova et al.	2024	International Immunopharmacology	Inflammation, mitochondrial and lysosomal dysfunction as key players in multiple sclerosis	No age-related analysis; disease-specific (multiple sclerosis) study without aging population data
**24**	Fernström et al.	2021	Translational Psychiatry	Blood-based mitochondrial respiratory chain function in major depressive disorder	No age-related analysis; psychiatric disease focus without aging biomarker comparison
**25**	Durhuus et al.	2020	Scientific Reports	Simvastatin improves mitochondrial respiration in peripheral blood cells	No age-related analysis; pharmacological intervention study without aging biomarker outcome
**26**	Mizuno et al.	2025	Environmental Health and Preventive Medicine	Peripheral blood mitochondrial DNA copy number as a predictor of	No age-related analysis; metabolic liver disease focus without aging population biomarker data

No.	Author(s)	Year	Journal	Title	Reason for Exclusion
				steatotic liver disease	
**27**	Lee et al.	2022	Frontiers in Endocrinology	Peripheral blood mononuclear cell mitochondrial copy number and adenosine in type 2 diabetes	No age-related analysis; focused solely on type 2 diabetes mechanism without aging biomarker outcome
**28**	Meng et al.	2025	European Journal of Medical Research	Integrated bioinformatics analysis unravels mitochondrial-immune crosstalk in inflammatory diseases	No age-related analysis; bioinformatics study without original blood aging data
**29**	Berry et al.	2024	Annals of the Rheumatic Diseases	Examining the biological pathways underlying clinical heterogeneity in rheumatoid arthritis	No age-related analysis; rheumatoid arthritis heterogeneity study without aging biomarker analysis

Exclusion criteria applied: (1) Mitochondrial function measured exclusively in non-blood tissues without parallel blood measurements; (2) Sample size < 15 participants without clear mechanistic justification; (3) No age-related analysis (cross-sectional age comparison or longitudinal follow-up); (4) Paediatric or disease-specific population without aging context; (5) Conference abstracts, editorials, letters, or opinion pieces without original data; (6) Review/meta-analysis articles without original primary data.
